# Renal atrophy following gated delivery of stereotactic ablative radiotherapy to adrenal metastases

**DOI:** 10.1016/j.phro.2021.09.001

**Published:** 2021-09-21

**Authors:** John R. van Sörnsen de Koste, Claire C. van Vliet, Famke L. Schneiders, Anna M.E. Bruynzeel, Berend J. Slotman, Miguel A. Palacios, Suresh Senan

**Affiliations:** Department of Radiation Oncology, Amsterdam University Medical Centers, location VUmc, de Boelelaan 1117, 1081 HV Amsterdam, The Netherlands

**Keywords:** SABR, Adrenal, Kidney, Organ at Risk, Renal dose constraints

## Abstract

Stereotactic ablative radiotherapy (SABR) planning for adrenal metastases aims to minimize doses to the adjacent kidney. Renal dose constraints for SABR delivery are not well defined. In 20 patients who underwent MR-guided breath-hold SABR in five daily fractions of 8–10 Gy, ipsilateral renal volumes receiving ≥20 Gy best correlated with loss of renal volumes, with median renal volume reduction being 6% (range: 3%-11%, 10th-90th percentiles). Organ function did not deteriorate in 18 patients, who had post treatment renal function tests available. This suggests that the ipsilateral renal volume receiving 20 Gy can be used as partial organ dose constraint for SABR to targets in the upper abdomen.

## 1 Introduction

The use of stereotactic ablative radiotherapy (SABR) is increasing in patients with oligometastatic disease, based on data emerging from both randomised phase II clinical trials and prospective registries [1]. It is important to minimize doses to organs at risk (OAR’s) during SABR delivery in order to avoid serious complications [2]. However, SABR to upper abdominal targets is challenging due to the close proximity of OAR's such as the stomach, bowel and kidneys, all of which exhibit intra- and interfraction motion [3], [4], [5]. In addition, the adrenal glands are embedded in the perirenal fat and enclosed by the renal fascia [6].

The available renal OAR constraints were largely derived from published experience in children and adults treated using conventionally fractionated radiotherapy. Only recently has renal function data emerged from patients treated using SABR [7], [8]. After SABR to primary renal tumors to a dose of 42 Gy in three fractions, each 10 Gy increase in physical dose led to an exponential decline of 25% in glomerular filtration rates (GFR) [7]. For pancreatic tumors, the renal volumes receiving 5 Gy during SABR were significantly associated with changes in GFR, a finding related to steep dose fall-offs outside the pancreas [8]. However, SABR delivery during free breathing may lead to less accurate estimates of doses to a mobile OAR. We implemented breath-hold MR-guided SABR delivery to adrenal metastases, using daily on-table plan adaption in order to improve target coverage and meet OAR constraints [4]. This breath-hold approach ensures a more reproducible patient anatomy, thereby allowing for more accurate renal dose estimates. Studying regional volume effects may provide for radiotherapy dose guidelines for selective sparing of either structures, or geometrically defined sections of potential organ at risks [9]. The aim of the present study was to establish the relationship between delivered cumulative SABR doses and renal atrophy after adrenal SABR.

## 2 Material and methods

Details of our MR-guided SABR approach for adrenal tumors have been previously reported [4]. Briefly, a daily pre-treatment 3D MR-scan is acquired during a shallow-inspiration breath-hold of 17 s, with an image resolution of 1.6 mm × 1.6 mm (axial, in-plane) and 3.0 mm (slice-thickness). After rigid registration of the gross tumor volume (GTV) on the pre-treatment planning MR scan, OAR contours are propagated to the MR-of-the-day using deformable image registration. The GTV and OAR’s are manually edited as needed. A planning target volume (PTV) is created by adding a 3 mm isotropic margin to the breath-hold GTV. Daily fraction sizes typically range from 8 Gy to 10 Gy, and daily plan re-optimization is based on the anatomy-of-the-day [10]. Adapted plans meet OAR dose constraints, using an ‘optimized PTV’ (PTVopt) that excludes parts of adjacent OAR’s. Planning constraints for the ipsilateral kidney require that two-thirds of the renal volume should not receive a dose exceeding 18 Gy. Gated treatment delivery is performed using visual feedback provided to patients on an in-room monitor, which projects both the GTV and gating window boundary in sagittal plane in real-time.

### 2.1 Patient selection criteria

All patient data were accessed from an institutional review board approved database. Most patients underwent diagnostic abdominal or thoracic CT-scans at around 3–6 months following SABR. Patients with a minimal follow-up of 24 months were eligible for study. For study eligibility, follow-up CT-scans had to have an axial slice thickness ≤ 3 mm, and thoracic CT-scans that imaged at least the upper 60% of the ipsilateral kidney, excluding regions of atrophy. A total of twenty patients with adrenal tumors who had a post-SABR scan at ≥24 months, were eligible, with 16 patients having a 30 month follow-up CT-scan. Five patients received immunotherapy during treatment (patients 2, 9, 18–20). No patient received chemotherapy concurrently with adrenal SABR. Two patients had undergone chemo-radiation for a primary lung tumor within 3 months of adrenal SABR with Cisplatin/Pemetrexed (patient 8), and Carboplatin/Alimta (patient 14). Following SABR, two patients received chemotherapy with Doxorubicine/Olaratumab (patient 17) and Cisplatin (patient 7) at 6 and 12 months, respectively. All MR-scans, the delivered dose plans and planning contours, and follow-up CT-scans were imported into VelocityAI (Varian Medical Systems, Palo, Alto, CA) for analysis.

### 2.2 Geometric analysis

In a preliminary study, we studied changes in the volume and shape of ten individual kidneys in different respiratory phases of 4DCT scans acquired during free breathing in five patients from a previous motion study [5] (data not shown). After image co-registration, the kidney contours of each patient on inspiration and expiration phases of 4DCT showed a median Dice coefficient value of 0.99 (range: 0.97–0.99, min–max). The kidney volumes in inspiration and expiration had a mean difference of 0.1% ± 0.4% (1SD).

Baseline ipsilateral renal volumes (IRVref) for patients in the present study were manually contoured on breath-hold planning CT-scans performed at the time of simulation for adrenal SABR. The IRVref excluded the renal hilus and blood vessels. The IRV on follow-up CT-scans was rigidly 6D-matched using overlaid IRVref contours. Renal contours were then manually adjusted to fit IRV on follow-up CT-scans, and saved to the breath-hold CT-scan as new structure files. A built-in Boolean operator tool was used to compute and visualize the volumetric differences between IRVref and the post-treatment IRV. A second observer re-checked the above procedures for each patient. Post-treatment IRV’s imaged on CT-scans were analyzed at 6, 12, 18, 24, and 30 monthly time points.

### 2.3 Analysis of dose/volume endpoints

The total doses delivered to IRVref were derived using rigid dose summation of the daily-adapted 3D dose delivery plans, which had been stored in the planning system after each treatment. The baseline simulation MR-scan was used to reconstruct the total delivered IRVref doses. Contour files from the breath-hold CT-scan were propagated to the baseline MR-scan. IRV positions on MR-scans performed prior to daily treatments were matched to the IRVref position on the baseline MR-scan with the corresponding 3D dose plans, and saved in DICOM-coordinate system of the baseline MR-scan. Finally, saved 3D dose plans of all treatment fractions of the patient were summed to derive the following dose-volume-histogram parameters: the IRVref proportion in ≥10 Gy (DVr10Gy) up to DVr30Gy, in incremental step of 5 Gy.

### 2.4 Renal function tests

GFR results were retrieved from clinical patient records. GFR tests were derived from the Chronic Kidney Disease Epidemiology Collaboration equation, which is based on the measured blood creatinine adjusted by age, gender and race. Results were reported in estimated GFR (eGFR) in ml/min/1.73 m^2^ values. Pre-treatment eGFR values and eGFR at 24 months or later following SABR were compared.

### 2.5 Statistical analysis

Numerical data were analyzed using Excel (Microsoft, Redmond, USA), using two-tailed t-tests to compare paired and un-paired data sets. Descriptive statistics were used to review univariate distribution of a data set; skewness and kurtosis between −2 and +2 were considered acceptable values for normal distribution. Pearson’s r correlation test was used to determine relationship between variables, and regression analysis to derive the corresponding p-value. A p-value threshold of 0.05 was used to indicate statistical significance.

## 3 Results

The majority of patients were males (15), and mean age was 67 ± 10 years. Most had left-sided tumors (n = 14), and one patient presented with bilateral adrenal metastases (patient 10).

Most patients (75%) received a prescribed PTVopt dose of 50 Gy, delivered in five daily fractions of 10 Gy. The median PTVopt was 22 cm^3^ (range: 3–161 cm^3^, min–max), and median IRVref was 138 cm^3^ (range: 89–206 cm^3^, min–max). The median distance between adrenal PTVopt and IRVref was 0.5 mm (range: 0.0–12.6 mm, min–max), and this was similar for both right- and left-sided tumors (p > 0.75, unpaired *t*-test). IRVref on planning CT-scans were similar to corresponding IRV’s on baseline MR-scans (p > 0.53, paired *t*-test); the median Dice’s coefficient value was 0.99 (range: 0.96–1.00, min–max). Details are available in Supplementary Table S1.

Pre- and post-SABR eGFR values at ≥24 months were available for eighteen patients. No significant differences from baseline values were found (p = 0.08, paired *t*-test); mean pooled baseline eGFR was 75.3 ± 16.1 ml/min/1.73 m^2^ versus post-SABR eGFR of 71.3 ± 15.9 ml/min/1.73 m^2^.

**Fig. 1:**
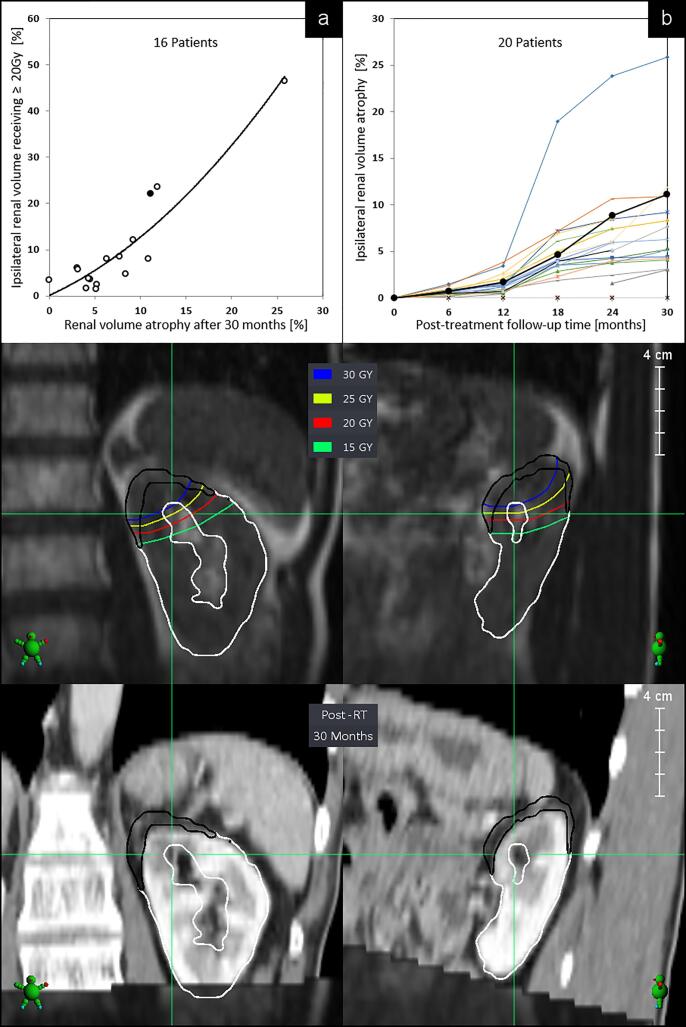
Changes in ipsilateral renal volumes for 16 patients in relation to the cumulative volume receiving ≥20 Gy (upper panel: a, trend line shown), and renal volumes for 20 patients over time (upper panel: b). Panels below show the baseline MR-simulation scan of a typical patient in coronal and sagittal planes, with superimposed renal doses. Contours in black indicate the area of loss of renal volume at the upper pole at 30 months post-treatment. The matched follow-up CT-scan is shown in the lower panels; data of the imaged patient is shown in both upper panels as filled black symbols.

Reductions in IRVref were observed adjacent to the adrenal tumor, with a median reduction of 6% (range: 3%-11%, 90th-10th percentiles). In patient 1, the maximum point dose in two-thirds of the ipsilateral renal volume showed 25 Gy as a result of a plan aiming to limit gastric doses. In this patient, the eGFR at 24 months post-SABR was identical to that at baseline, and the contralateral renal volume increased by ∼23%. A similar pattern of IRVref atrophy was observed in most patients, with mild atrophy seen in the first 12 months, followed by a more pronounced change between 12 and 24 months (p < 0.01, paired *t*-test) than at a later time point (Fig. 1, panel: b). Dose-volume-histograms revealed that IRVref proportions treated to ≥20 Gy (DVr20Gy) best predicted for reduction in IRVref (Fig. 1, panel: a; Supplementary Table S1). A significantly correlation (Pearson’s r test, (p < 0.01)) was seen between DVr20Gy and IRVref atrophy at 18-, 24- and at 30-months post-SABR, with correlation r values of 0.76, 0.81, and 0.92, respectively. Finally, exclusion of patients who had received a dose of <50 Gy did not alter our findings.

## 4 Discussion

The preferred dose for adrenal SABR in patients with oligometastatic disease is a BED_10Gy_ of at least 100 Gy, as this correlates with long-term tumor control [11], [12]. Despite the close proximity of the kidneys and adrenals, this study revealed only limited IRVref atrophy after delivery of high-dose breath-hold SABR to adrenal metastases. This finding may reflect the use of 3 mm planning margins during SABR delivery during repeat breath-holds [4]. We also observed that ipsilateral renal volumes receiving a dose of ≥20 Gy dose (DVr20Gy) best predicted late IRV atrophy. The use of larger margins will increase the irradiated kidney volume, but we believe the relationship between DVr20Gy and atrophy post-SABR will remain valid. The patterns of atrophy were similar in all patients studied, and changes were most pronounced between 12 and 24 months.

The ipsilateral kidney dose constraints used for planning adrenal SABR at our center were that the maximum point dose to two-thirds of the ipsilateral renal volume not exceed 18 Gy. Non-uniform dose distributions are routinely used with MR-guided breath-hold SABR delivery, and findings of the present study suggest that techniques which minimize renal volumes treated to ≥20 Gy may be preferred. In this analysis, we performed rigid dose summation from each SABR fraction in order to estimate the total delivered ipsilateral renal dose. We believe that this approach is justified as the shape and volume of kidneys during breathing are unchanged on 4DCT studies, and as baseline MR-based and simulation CT-based renal volumes in all study patients were comparable (Supplementary Table S1).

Contralateral renal hypertrophy was only observed in patient 1 who had significant ipsilateral renal atrophy after 46% of the renal volume had received 20 Gy (Fig. 1; panel a). However, in two other patients in whom approximately 23% of the ipsilateral renal volume received 20 Gy, the contralateral renal volumes remained unchanged.

The renal volume-function effects observed in this study are consistent with that reported by others. Following large-field conventional radiotherapy to patients with cervical or endometrial cancer, delivered in once-daily fractions of 1.8 Gy, a strong correlation was reported between the volumes of both kidneys receiving 20 Gy and changes in post-treatment eGFR [13]. A study of complications associated with treatment of abdominal vascular aneurysms also observed a significant association between total renal volume and eGFR [14].

A limitation of our study is that standardized follow-up imaging of the upper abdomen was not performed for all patients. However, only limited volumetric changes were observed in our cohort (median 6%), and these were restricted to renal volumes adjacent to the adrenal metastasis, making measurable changes in eGFR unlikely. Stable eGFR values were also reported after robotic SABR using implanted fiducials in ten patients with renal carcinoma [15], suggesting that gated delivery contributes to preservation of renal function.

Although the access to MR-Linacs is presently limited, our findings are relevant to delivery of breath-hold SABR on CT-Linacs, and gating on CyberKnife units.

In conclusion, our findings suggest a strong correlation between ipsilateral renal volumes receiving ≥20 Gy during breath-hold SABR delivery, and subsequent loss of renal volume. This suggests that use of a partial organ dose constraint of 20 Gy is justified for gated SABR delivery to the adrenals and primary renal tumors.

## 5 Financial support

No industry funding was received for the planning and conduct of this study. The content of the work is solely the responsibility of the authors.

## Declaration of Competing Interest

The authors declare the following financial interests/personal relationships which may be considered as potential competing interests: The Department of Radiation Oncology at the Amsterdam University Medical Center (location VUmc) has funded research agreements with ViewRay Inc. and Varian Medical Systems. No industry funding was received for the planning and conduct of this study. The content of the work is solely the responsibility of the authors. Details of each author below: John R. van Sörnsen de Koste, Claire C. van Vliet and Famke L. Schneiders have nothing to disclose. Anna M.E. Bruynzeel reports grants from ViewRay Inc, during the conduct of the study; personal fees from Viewray, Inc., outside the submitted work. Berend J. Slotman reports grants from Varian Medical Systems, grants from ViewRay, personal fees from ViewRay, outside the submitted work. Miguel A. Palacios reports personal fees from ViewRay, Inc., outside the submitted work. Suresh Senan reports grants from ViewRay Inc., during the conduct of the study; grants from Varian Medical Systems, grants and personal fees from AstraZeneca, personal fees from MSD, personal fees from Beigene, outside the submitted work.

## References

[1] Correa R.J.M., Palma D.A. (2021). Should stereotactic ablative body radiotherapy be the standard of care in oligometastatic cancer?. Lancet Oncol.

[2] Palma D.A., Olson R., Harrow S., Gaede S., Louie A.V., Haasbeek C. (2019). Stereotactic ablative radiotherapy versus standard of care palliative treatment in patients with oligometastatic cancers (SABR-COMET): a randomised, phase 2, open-label trial. Lancet.

[3] van Sörnsen de Koste J.R., Palacios M.A., Chen H., Schneiders F.L., Bruynzeel A.M.E., Slotman B.J. (2020). Changes in gastric anatomy after delivery of breath-hold MR-guided SABR for adrenal metastases. Radiother Oncol.

[4] Palacios M.A., Bohoudi O., Bruynzeel A.M.E., van Sörsen de Koste J.R., Cobussen P., Slotman B.J. (2018). Role of daily plan adaptation in MR-guided stereotactic ablative radiation therapy for adrenal metastases. Int J Radiat Oncol Biol Phys.

[5] van Sörnsen de Koste JR, Senan S, Kleynen CE, Slotman BJ, Lagerwaard FJ. Renal mobility during uncoached quit respiration: an analysis of 4DCT scans. Int J Radiat Oncol Biol Phys 2006;64:799–803. 10.1016/j.ijrobp.2005.09.012 16298498

[6] Avisse C., Marcus C., Patey M., Ladam-Marcus V., Delattre J.-F., Flament J.-B. (2000). surgical anatomy and embryology of the adrenal glands. Surg Clin North Am.

[7] Siva S., Jackson P., Kron T., Bressel M., Lau E., Hofman M. (2016). Impact of stereotactic radiotherapy on kidney function in primary renal cell carcinoma: establishing a dose–response relationship. Radiother Oncol.

[8] Verma V., Bhirud A.R., Denniston K.A., Bennion, Lin C. (2017). Quantification of renal function following stereotactic body radiotherapy for pancreatic cancer: secondary dosimetric analysis of a prospective clinical trial. Radiat Oncol.

[9] Casares-Magaz O., Moiseenko V., Witte M., Rancati T., Muren L.P. (2020). Towards spatial representations of dose distributions to predict risk of normal tissue morbidity after radiotherapy. Phys Imaging Radiat Oncol.

[10] Bohoudi O., Bruynzeel A.M.E., Senan S., Cuijpers J.P., Slotman B.J., Lagerwaard F.J. (2017). Fast and robust online adaptive planning in stereotactic MR-guided adaptive radiation therapy (SMART) for pancreatic cancer. Radiother Oncol.

[11] Chance W.W., Nguyen Q., Mehran R., Welsh J.W., Gomez D.R., Balter P. (2017). Stereotactic ablative radiotherapy for adrenal gland metastases: Factors influencing outcomes, patterns of failure, and dosimetric thresholds for toxicity. Pract Radiat Oncol.

[12] Desai A., Rai H., Haas J., Witten M., Blacksburg S., Schneider J.G. (2015). A retrospective review of CyberKnife stereotactic body radiotherapy for adrenal tumors (primary and metastatic): Winthrop University Hospital experience. Front Oncol.

[13] Kunogi H., Yamaguchi N., Terao Y., Sasai K. (2021). Dosimetric predictors of nephrotoxicity in patients receiving extended-field radiation therapy for gynecologic cancer. Radiat Oncol.

[14] Martin-Gonzalez T., Pinçon C., Maurel B., Hertault A., Sobocinski J., Spear R. (2015). Renal outcomes following fenestrated and branched endografting. Eur J Vasc Endovasc Surg.

[15] Senger C., Conti A., Kluge A., Pasemann D., Kufeld M., Acker G. (2019). Robotic stereotactic ablative radiotherapy for renal cell carcinoma in patients with impaired renal function. BMC Urol.

