# Supplementary material for: Renal atrophy following gated delivery of stereotactic ablative radiotherapy to adrenal metastases
Source: Phys Imaging Radiat Oncol. 2021 Sep 21;20:1–4. doi: 10.1016/j.phro.2021.09.001 (PMC8473532; doi:10.1016/j.phro.2021.09.001)
Supplement: Supplementary Data 1 [file mmc1.docx]

**Supplementary Table S1**: Overview of dosimetric parameters in individual patients. Significant correlations were observed between absorbed renal volume dose (DVr) and the ipsilateral volume atrophy observed post-SABR treatment at 24 and 30 months, respectively.

| Patient | PTVopt  (total dose) | PTVopt  (location) | PTVopt  (cm^3^) | IRVref planning CT-scan (cm^3^) | IRV simulation MR-scan (cm^3^) | Minimum distance in 3D: PTVopt-IRVref (mm) | IRVref atrophy at 24 months | IRVref atrophy at 30 months | DVr10Gy | DVr15Gy | DVr20Gy | DVr25Gy | DVr30Gy |
| --- | --- | --- | --- | --- | --- | --- | --- | --- | --- | --- | --- | --- | --- |
|  |  |  |  |  |  |  |  |  |  |  |  |  |  |
| 1 | 50Gy | Left | 34.8 | 172.1 | 170.7 | 0.0 | 23.8% | 25.9% | 61.4% | 54.8% | 46.4% | 33.5% | 22.4% |
| # 2 | 50Gy | Left | 16.6 | 102.4 | 102.7 | 0.0 | 8.9% | 11.1% | 41.3% | 30.8% | 22.1% | 15.8% | 11.0% |
| 3 | 50Gy | Left | 33.0 | 149.9 | 149.4 | 0.0 | 1.6% | 3.1% | 12.4% | 9.1% | 6.1% | 4.4% | 3.1% |
| 4 | 50Gy | Left | 87.4 | 148.9 | 147.4 | 0.0 | 8.5% | 9.2% | 28.4% | 19.2% | 12.2% | 6.9% | 3.6% |
| 5 | 50Gy | Left | 22.0 | 133.9 | 134.5 | 4.6 | 2.5% | 3.1% | 23.8% | 12.5% | 5.8% | 2.6% | 1.0% |
| 6 | 50Gy | Left | 19.0 | 152.5 | 153.7 | 4.1 | 4.0% | 4.3% | 25.1% | 10.5% | 3.9% | 0.0% | 0.0% |
| 7 | 50Gy | Left | 17.3 | 89.4 | 91.7 | 0.0 | 5.0% | 7.7% | 27.8% | 15.9% | 8.6% | 4.6% | 2.2% |
| 8 | 50Gy | Left | 16.3 | 135.0 | 136.9 | 1.3 | 7.4% | n.a | 24.7% | 11.8% | 6.6% | 4.2% | 2.7% |
| 9 | 50Gy | Left | 19.9 | 160.9 | 159.4 | 1.7 | 6.0% | n.a | 53.3% | 38.0% | 20.0% | 10.9% | 6.4% |
| 10 | 50Gy | Left | 3.0 | 128.5 | 126.8 | 12.6 | 0.0% | n.a | 2.5% | 0.8% | 0.1% | 0.0% | 0.0% |
|  | 50Gy | Right | 16.4 | 119.9 | 118.2 | 4.6 | 5.2% | n.a | 16.9% | 9.6% | 5.0% | 2.3% | 1.0% |
| 11 | 50Gy | Right | 68.9 | 107.9 | 106.6 | 0.0 | 10.9% | n.a | 51.6% | 37.1% | 23.1% | 14.4% | 9.0% |
| 12 | 50Gy | Right | 20.6 | 191.5 | 190.6 | 0.8 | 7.5% | 8.4% | 16.0% | 8.8% | 4.8% | 2.7% | 1.5% |
| 13 | 50Gy | Right | 49.4 | 137.7 | 137.3 | 0.8 | 4.4% | 4.4% | 8.1% | 5.6% | 3.7% | 2.5% | 1.6% |
| 14 | 50Gy | Right | 16.7 | 125.2 | 127.1 | 6.2 | 3.8% | 4.1% | 5.9% | 3.3% | 1.7% | 0.7% | 0.1% |
| 15 | 50Gy | Right | 100.8 | 139.4 | 140.2 | 0.3 | 6.0% | 6.3% | 17.7% | 11.7% | 8.1% | 5.8% | 4.2% |
| 16 | 40Gy | Left | 6.5 | 108.9 | 109.2 | 8.7 | 0.0% | 0.0% | 32.1% | 11.4% | 3.5% | 0.9% | 0.1% |
| 17 | 40Gy | Right | 161.3 | 136.0 | 136.3 | 0.0 | 3.4% | 11.9% | 40.3% | 31.0% | 23.6% | 17.5% | 11.9% |
| 18 | 30Gy | Left | 83.4 | 206.4 | 206.5 | 0.0 | 10.6% | 10.9% | 25.3% | 15.7% | 8.1% | 3.1% | 0.1% |
| 19 | 24Gy | Left | 32.3 | 188.6 | 186.1 | 0.0 | 4.3% | 5.2% | 21.8% | 7.7% | 2.4% | 0.1% | 0.0% |
| 20 | 24Gy | Left | 35.8 | 150.2 | 148.9 | 0.5 | 3.7% | 5.1% | 21.4% | 7.3% | 1.4% | 0.0% | 0.0% |
|  |  |  |  |  |  |  | ^##^Correlation ( r ) | | +0.64 | +0.75 | **+0.81** | +0.79 | +0.76 |
|  |  |  |  |  |  |  | Correlation ( r ) | | +0.77 | +0.91 | **+0.92** | +0.91 | +0.88 |

**Footnotes (table 1):**

# Patient highlighted in figure 1

## Pearson’s r correlation coefficient test

Abbreviations: PTVopt = Optimized planning target volume; IRVref = Baseline ipsilateral renal volume; DVr10Gy = the proportion of IRVref that received ≥ 10Gy; CT = computed tomography; MR = magnetic resonance imaging.
